# Onsite Graywater Treatment in a Two-Stage Electro-Peroxone Reactor with a Partial Recycle of Treated Effluent

**DOI:** 10.1021/acsestengg.1c00240

**Published:** 2021-10-11

**Authors:** Léopold Dobelle, Seungkyeum Kim, Axl X. LeVan, Hugo Leandri, Michael R. Hoffmann, Clément A. Cid

**Affiliations:** † Department of Environmental Science and Engineering, California Institute of Technology, 1200 E California Blvd, Pasadena, California 91125, United States; ‡ Department of Chemical Engineering, California Institute of Technology, 1200 E California Blvd, Pasadena, California 91125, United States; § Department of Chemistry, California Institute of Technology, 1200 E California Blvd, Pasadena, California 91125, United States

**Keywords:** electro-peroxone, graywater recycling, ozone, hydrogen peroxide

## Abstract

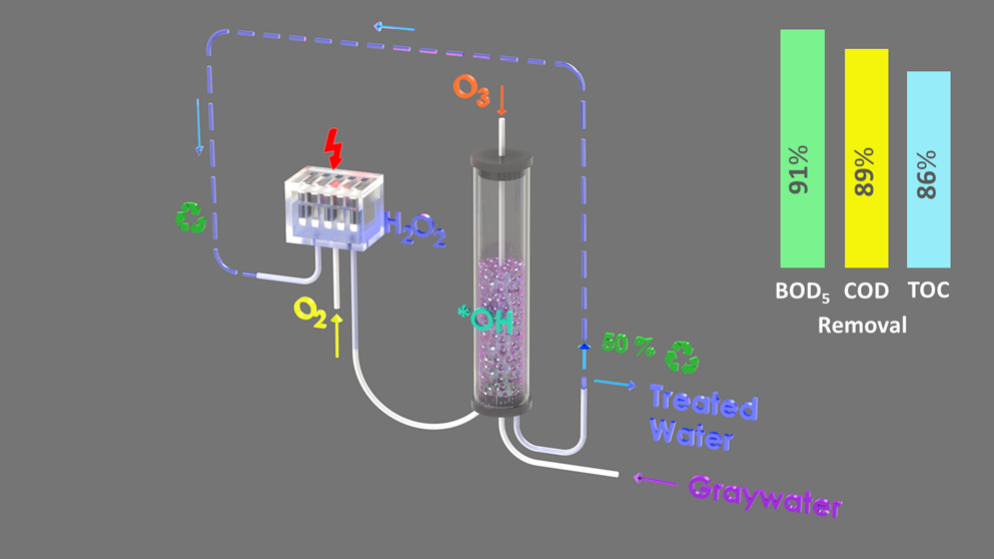

The efficacy of an uncoupled electro-peroxone (E-peroxone) prototype reactor system for the treatment of synthetic graywater is determined. The two-stage E-peroxone process integrates ozonation with the *in situ* production of hydrogen peroxide (H_2_O_2_) in a first stage reactor before ozone (O_3_) is converted via the peroxone reaction to a hydroxyl radical (^•^OH). The two-stage prototype reactor system allows for the generation of H_2_O_2_ via cathodic oxygen reduction in the first-stage reactor before mixing with O_3_ in the second-stage reactor. This approach prevents the degradation of polytetrafluoroethylene (PTFE) coated carbon cathodes by ^•^OH that takes place in a single well-mixed reactor that combines electrochemical peroxide generation with O_3_. The dosage of H_2_O_2_ into the second-stage reactor is optimized to enhance graywater treatment. Under these conditions, the uncoupled E-peroxone system is capable of treating synthetic graywater with an initial chemical oxygen demand (COD_0_) of 358 mg O_2_/L, a total organic carbon (TOC_0_) of 96.9 mg/L, a biochemical oxygen demand (BOD_0_) of 162 mg O_2_/L, and a turbidity of 11.2 NTU. The two-stage electro-peroxone system can reduce the initial COD_0_ by 89%, the TOC_0_ by 91%, BOD_0_ by 86%, and the turbidity by 95% after 90 min of treatment. At this performance level, the reactor effluent is acceptable for discharge and for use in nonpotable applications such as toilet-water flushing. A portion of the effluent is recycled back into the first-stage reactor to minimize water consumption. Recycling can be repeated consecutively for four or more cycles, although the time required to achieve the desired H_2_O_2_ concentration increased slightly from one cycle to another. The two-stage E-peroxone system is shown to be potentially useful for onsite or decentralized graywater treatment suitable for arid water-sensitive areas.

## 1 Introduction

Climate change has increased water scarcity in many parts of the world, leading to the need for development of new practices for water supply management.^1,2^ Onsite water reuse is one of the key opportunities to increase water supply without a detrimental impact on the environment.^3^ Important steps in this direction have been taken through the development of nonsewered sanitation systems with low- or near-zero water consumption competing with large scale wastewater treatment plants.^4,5^ A complementary approach is the treatment and reuse of graywater. This new paradigm provides a steady water supply that is not influenced by seasonal availability or need.^6^ Furthermore, the level of desired treatment can range from treating graywater for reuse in low contact systems (e.g., as flushing water) or as a first-step treatment to convert processed water into potable water.^1,2^

In order to achieve a high level of water quality for reuse as described in the recommended NSF 350 standard,^7^ graywater treatment systems often employ the same processes as used in large-scale wastewater treatment plants. In general, the treatment train starts either with primary sedimentation or multimedia filtration; these steps are then followed by fixed-bed biological treatment and chemical disinfection.^8^ Even though this approach has proven to be effective, the biological treatment requires a residence time ranging from 5 to 24 h^9^ in a large-volume bioreactor. Advanced graywater treatment systems capable of reducing the organic load and disinfecting the product water without biological treatment may allow for a much smaller size for a household graywater recycling system.

Advanced oxidation processes (AOPs) primarily rely on *in situ* generation of a hydroxyl radical (^•^OH) as the primary oxidant due to its very high reduction potential and related reactivity as a one-electron oxidant of susceptible organic and inorganic electron donors (*E*°(^•^OH/H_2_O) = 2.80 V vs SHE). Physico-chemical methods for hydroxyl radical production include ultrasonic radiation or sonolytic ozonation,^10^ UV/H_2_O_2_ and UV/O_3_ photolysis, or utilizing the O_3_/H_2_O_2_ peroxone reaction.^11^ However, these methods are limited by their operational costs. In the case of hydrogen peroxide, storage of high concentrations (e.g., ≥30% by weight of H_2_O_2_) requires special hazardous chemical precautions. Hydrogen peroxide is susceptible to autocatalytic decomposition into oxygen and water, which may lead to an explosion in an unvented storage container.

To avoid the high cost of H_2_O_2_, the electro-peroxone process (E-peroxone) can be used to produce H_2_O_2_ onsite via the electrochemical reduction of oxygen. Since both ozone and H_2_O_2_ are generated onsite with oxygen, problems associated with reagent storage are avoided.

Combining electrochemical H_2_O_2_ production using carbon cathodes coupled with O_3_ also generated onsite in a single reactor has been investigated by Wang,^12^ but the short lifetimes of carbon cathodes in the presence of ^•^OH make the single reactor approach challenging for practical applications. This is particularly true for small-scale treatment of wastewater in onsite, semiautonomous units where component replacement should be minimized.^13^ In comparison to the single-step E-peroxone process, H_2_O_2_ production is separated from the actual peroxone reaction chamber in order to increase the lifetime of the carbon cathodes from attack by ^•^OH. The quality of the effluent from the dual-chamber reaction system is designed to be suitable for either discharge, recycling as toilet flushing water, or returned into the H_2_O_2_ production chamber as a source water. The system as tested is shown to be suitable for either a single-pass treatment sequence or for the continuous reuse of treated graywater as an influent for the H_2_O_2_ generation step. The goals of the reaction system are to meet the requirements of the standards established by NSF 350/350– for effluent quality terms of COD, TOC, BOD, pH, and turbidity for either safe discharge into receiving waters or reuse for nonpotable water applications such as toilet and urinal flushing.

## 2 System Design

### 2.1 Overall Design

**Figure 1:**
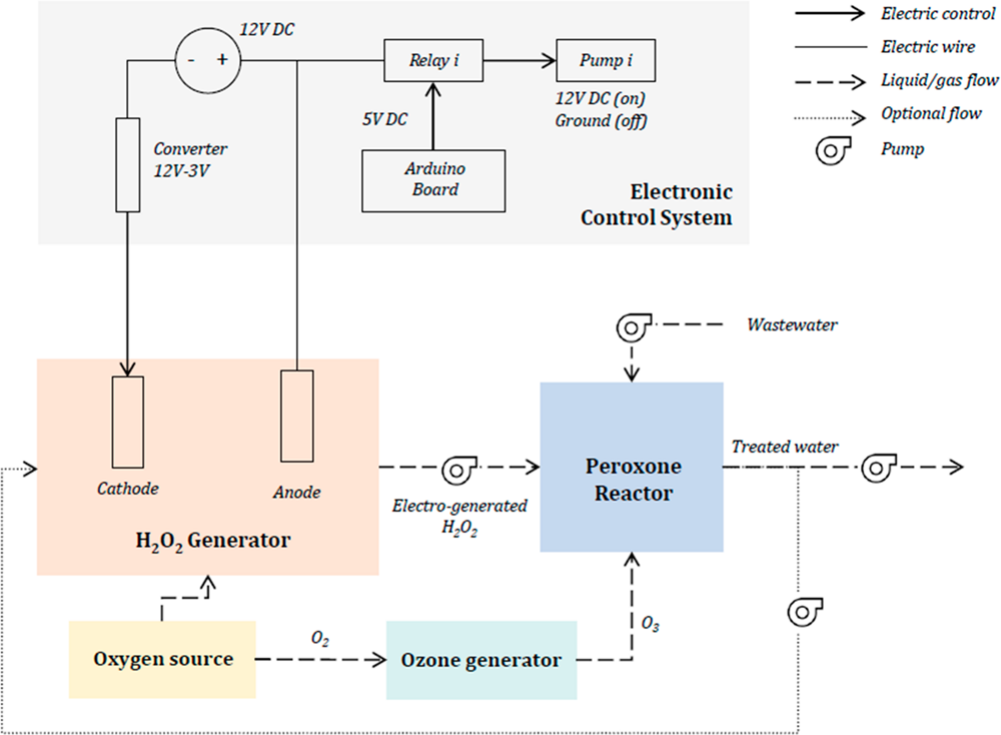
Schematic diagram of an integrated electro-peroxone reactor system. A single power supply provides the applied potential power for the electrolysis and to run the mechanical components needed for a continuous-flow reaction system.

The electro-peroxone system developed in this study consists of two separate chambers as shown in Figure 1. The first chamber is an electrochemical H_2_O_2_ generator, and the second chamber is the reactor for the peroxone reaction leading to ^•^OH production. A laboratory-scale prototype is shown in Figure S1 (Supporting Information). The two chambers are connected using flexible silicone tubing (5 mm ID × 7 mm OD, Uxcell, Hong Kong, China) to transfer the electrochemically generated H_2_O_2_ from the first reactor to the peroxone reactor using a peristaltic pump (INTLLAB, Shenzhen Jiashi Technology Co. Ltd., Shenzhen, China). Influent water (e.g., synthetic graywater) is pumped into the peroxone reactor, where it was combined with O_3_ and electrochemically generated H_2_O_2_. Finally, the treated water is either collected or recycled for use in the following cycle of treatment. Both chambers have collection ports for sampling.

### 2.2 Electrochemical H_2_O_2_ Generation

The H_2_O_2_ generator is a custom-designed 500 mL reactor made from ABS-based plastic (VisiJet Armor (M2G-CL; MJP)) that is 3D-printed using a ProJet MJP 2500 Plus (3D SYSTEMS, Rock Hill, SC). A titanium O_2_ diffuser is placed along the center line and at the bottom of the reactor at a flow rate of 0.8 standard cubic feet per hour (SCFH). The electrode pair is placed above the O_2_ diffuser in a 3D-printed 110 mm × 70 mm × 105 mm ABS-based housing, which covers the edges of the electrodes in prevent electrical shortages and to increase the mechanical stability of the electrolysis unit (Figure S2, Supporting Information). The electrolysis cell consists of three polytetrafluoroethylene (PTFE) coated carbon cathodes (CP75T carbon fiber paper, AVCARB MATERIAL SOLUTIONS, Lowell, MA) placed in a sandwich configuration between two IrTaO-TiO_*x*_ anodes (Nanopac, Korea). A constant electrical potential of +3 V is applied between the cathodes and the anodes during H_2_O_2_ electrolysis. 50 mM Na_2_SO_4_ (Macron Fine Chemicals, Center Valley, PA) in deionized water (Milli-Q, Millipore) is initially used as the electrolyte. H_2_O_2_ is generated at the cathodes, whereas mainly oxygen gas is produced at the anodes as a half reaction of water splitting. In the subsequent treatment cycles, treated graywater is used as the electrolyte.

### 2.3 Peroxone Reactor

The peroxone reactor is a customized 1-L cylinder with two liquid ports and one gas infuser. The influent (i.e., synthetic graywater) is added directly from an external waste container, while H_2_O_2_ is transferred from the H_2_O_2_ generator and introduced at the bottom of the peroxone reactor using flexible tubing. An external O_3_ generator (CNC6390-1V, Eleoption) with an average power consumption of 65 W is used to convert oxygen to O_3_ with a 5% efficiency, delivering 71.5 mg/L of O_3_ to the peroxone reactor during the cycle treatment. The O_2_/O_3_ diffuser at 0.8 SCFH was placed slightly above the bottom of the reactor. Mixing in the reactor is achieved by the turbulence generated by the flow of O_2_/O_3_. Treated wastewater is pumped out from the bottom of the cylinder to an external storage container.

### 2.4 Electronics and Pumps

The entire system is powered by a 12 V DC, 0–30 A power supply (Supermight). The output voltage is converted to 3 V using an adjustable DC–DC converter (DROK LM2596) to drive the H_2_O_2_ generator. The output voltage is continuously monitored and displayed using a MCIGICM 0.28″ LED Voltmeter Ammeter.

Liquid flow in the system is controlled by five identical INTLLAB peristaltic pumps of 3 mm ID × 5 mm OD, powered by the 12 V DC, 0–30 A power supply. The flow from each pump is controlled at 100 mL/min by five YOUNGNEER 5 V Relays controlled by an ARDUINO UNO R3 board.

## 3 Methods

### 3.1 Treatment Sequence

The system described in section 2 was used in all of the treatment tests presented in this study. A full test sequence started with H_2_O_2_ generation using 0.8 SCFH of O_2_ and 3 V of applied potential at the electrodes of the H_2_O_2_ generator. The H_2_O_2_ generation step lasted until the desired concentration of H_2_O_2_ (∼2.25 abs or 4.8 mM) was reached (between 60 to 90 min). A 250 mL solution of the electrochemically generated H_2_O_2_ was introduced into the peroxone reactor in three injections of equal volume, each at 0, 10, and 20 min, unless noted otherwise. O_2_ was converted to O_3_ by the O_3_ generator and continuously introduced into the peroxone reactor at 0.8 SCFH until the end of treatment. Aliquots of graywater were collected for chemical and physical analysis every 10 min for the first 60 min of treatment and at the end of the treatment for the last 90 min.

### 3.2 Consecutive Runs

The first treatment sequence was conducted using 50 mM Na_2_SO_4_ electrolyte in 500 mL of DI water for H_2_O_2_ generation. Treated graywater was used as the subsequent electrolyte for the following treatment cycles. In this case, 440 mL of the treated graywater was added back into the H_2_O_2_ reactor before electrolytic H_2_O_2_ generation. The submerged electrode surface area was kept constant in the H_2_O_2_ chamber. Two 20 mL glass vials were immersed in the chamber to compensate for the volume loss caused by sampling during treatment. The duration of the electrolysis of H_2_O_2_ was adapted to reach a concentration of ∼4.8 mM H_2_O_2_. Following the generation of H_2_O_2_, all consecutive steps were identical to the full system treatment sequence as described in section 3.1. This process was repeated until the fourth treatment sequence was achieved. The reactor and associated tubing were flushed with 500 mL of DI water between each run.

### 3.3 Cyclic Voltammetry

Cyclic voltammetry (CV) was performed in a 50 mM Na_2_SO_4_ solution in a four-necked flask, employing a three-electrode configuration that was connected to a Bio-Logic VSP-300 Potentiostat (Seyssinet-Pariset, France). The pH of the electrolyte was 5.61. The working electrode was a 1 cm × 1 cm PTFE-coated carbon paper electrode exposed on a single side and covered on the backside with epoxy. A CHI 151 Hg/HgSO_4_ electrode (CH Instruments, Austin TX, USA) was used as the reference electrode, while a platinum counter electrode and an outlet for gases occupied the third neck of the flask. The fourth neck was used as an inlet for nitrogen (N_2_) or oxygen (O_2_) gas purging. N_2_ or O_2_ was purged into the flask for saturation for 30 min before CV and then constantly purged throughout the CV scans. Either gas was bubbled through another 50 mM Na_2_SO_4_ solution before introduction to the flask to reduce evaporation. CV was performed at scan rate of 10 mV/s in the potential range of −1.0 to 0.0 V.

### 3.4 Cathode Materials

The H_2_O_2_ production rates over 60 min using different carbon-based materials were determined (Table S1, Supporting Information). Chemical compatibility and the cost of materials were also considered. Each cathode material had the same surface area (8 cm^2^) in contact with 400 mL of a solution of Na_2_SO_4_ at 50 mM. The anode was a composite IrTaO-TiO_*x*_ electrode (Nanopac, Korea). Oxygen was supplied at a constant flow rate of 1.7 SCFH.

### 3.5 Sample Collection and Characterization

First, 10 mL samples were collected 10 cm from the bottom of the peroxone reactor using a 25 mL pipet and were briefly stored at room temperature (21 ± 1 °C) before being analyzed for turbidity, [H_2_O_2_], COD, pH, and TOC. Unless noted otherwise, the measurements were performed in triplicate.

#### 3.5.1 Turbidity

Turbidity was measured using the HI93414 Turbidity meter (Hanna Instruments, Woonsocket, USA) following the method recommended by the manufacturer.

#### 3.5.2 [H_2_O_2_]

A 0.5 mL sample was combined with an equal volume of a titanium oxalate solution as per Sellers.^14^ The 407 nm absorbance of the resulting mix was measured by UV–vis spectroscopy using a Nanodrop 2000c spectrophotometer (Thermo Scientific, Waltham, USA). The system was blanked with Milli-Q water beforehand.

#### 3.5.3 Chemical Oxygen Demand (COD)

After appropriate dilution, COD was measured by colorimetry following Hach Method 8000 with low-range 3–150 mg O_2_/L COD vials and a DR 900 Colorimeter (Hach, Loveland, USA). Interferences on COD measurement due to residual H_2_O_2_ in the peroxone reactor were monitored and stayed under 10 mg O_2_/L.

#### 3.5.4 pH Measurement

pH was determined using an Orion Star A215 pH/conductivity meter (Thermo Scientific, Waltham, USA) connected to an Orion 8157BNUMD Ross Ultra pH/ATC Triode (Thermo Scientific, Waltham, USA).

#### 3.5.5 Total Organic Carbon (TOC) and Total Inorganic Carbon (TIC)

In addition to COD measurements, the Total Organic Carbon (TOC) and Total Inorganic Carbon (TIC) concentrations in the synthetic graywater samples were measured over the course of treatment using a TOC analyzer (OI Analytical Model 1030W, College Station, TX). The TOC content was obtained indirectly by subtracting the measured TIC content from the measured total carbon (TC) content. While TIC was quantified in the gas produced by phosphoric acid (5% v/v, Fisher Scientific, Hampton, NH) treatment, TC was quantified by oxidation of all the existing carbon in the sample with Na_2_S_2_O_8_ (10% w/v, Acros Organics, Fair Lawn, NJ).^15^ The sample was diluted at a 1:4 ratio with Milli-Q water prior to analysis, except for the last extract obtained at 90 min due to its very low TOC content.

#### 3.5.6 Total Nitrogen (TN) and Total Phosphorus (TP)

Total nitrogen (TN) and total phosphorus (TP) of the synthetic graywater were determined by colorimetry using a Hach DR 900 colorimeter (Hach, Loveland, USA) at the beginning and at the end of the treatment. For the TN measurement, Hach Method 10071 was used with Test ‘N Tube Low Range Total Nitrogen Reagent Set. For the TP measurement, Hach method 8190 was used with the Test ‘N Tube Low Range Total Phosphate Reagent Set.

#### 3.5.7 Biochemical Oxygen Demand (BOD)

Biochemical Oxygen Demand (BOD) was measured after a 5-day incubation period following Standard Method 5210B^16^ using an Accumet XL40 Dissolved Oxygen Meter (Fisher Scientific, Waltham, MA) connected to an Orion BOD probe (Thermo Scientific, Waltham, MA). The samples collected after treatment (90 min) were bubbled with air for approximately 5 min after dilution and prior to measuring the dissolved oxygen.

### 3.6 Graywater Synthesis

The synthetic graywater used in this study (Table S2, Supporting Information) was adapted from the NSF 350/350-1 standard.^17^ A 10-L batch of synthetic graywater was prepared in a 30-L container by dissolving the components in tap water, except for deodorant and toothpaste. A premix solution of deodorant and toothpaste was prepared separately in 450 mL of tap water at 65 °C and mixed at 700 rpm for 20 min and then added to the container. Finally, the synthetic graywater solution was mixed with a DLH overhead stirrer (Velp Scientifica, Usmate, Italy) at 2000 rpm for 20 min and settled for 10 min before use. Synthetic graywater from the same batch was used for each consecutive testing. The graywater was characterized and found to be consistently within the range of water matrices described in the NSF 350/350-1 standard (see Table S3, Supporting Information).

## 4 Results and Discussion

### 4.1 Single Run Testing

#### Removal of COD and TOC

**Figure 2:**
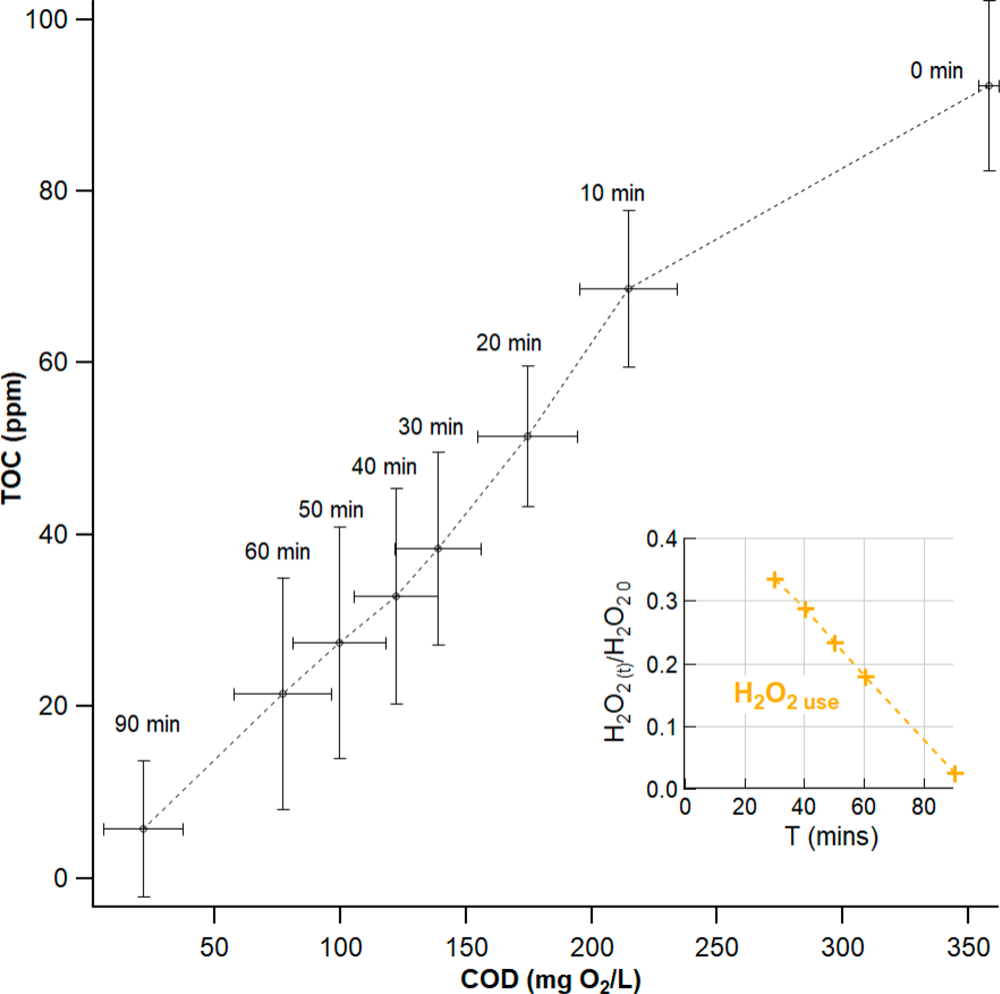
Co-evolution of TOC and COD concentrations in the peroxone reactor. Electrochemically generated H_2_O_2_ was added to the reactor in equal volumes after 0, 10, and 20 min of E-peroxone treatment. (Inset: concomitant evolution of [H_2_O_2_] in the peroxone reactor during treatment.) Error bars represent ± standard deviation from the mean.

A decrease in COD and TOC concentrations in the bulk solution of the peroxone reactor was observed during each run (Figure 2) following the treatment sequence described in section 3.1. Graywater had an average initial COD level of 358.5 ± 3.8 mg O_2_/L and reached a COD level of 21.25 ± 15.70 mg O_2_/L at the end of the 90 min treatment run. The average percent COD removal was 94% in solution and 89% when adjusted for dilution. In addition to observing COD removal, the TOC of the solution was degraded by 92% (86% adjusted for dilution) during the E-peroxone process with starting TOC concentrations of 96.95 ± 9.84 mg/L and final TOC concentrations of 7.29 ± 7.86 mg/L. As a comparison, conventional ozonation was performed by substituting H_2_O_2_ with Milli-Q water added to the system in the same injection patterns (0 min, 10 min, and 20 min), and we observed both COD and TOC removals of only 57% and 43%, respectively. The large difference between injection of H_2_O_2_ solution or Milli-Q water confirms that the E-peroxone treatment was more effective at oxidizing and mineralizing organics than ozonation alone.^11^

The decrease of COD and TOC concentration over time was not linear (Figure 2). COD and TOC decreased by an average of 34% and 27% during the first 10 min of the process, while this removal was of approximately 19% and 20% during the next 10 min increments, with limited effect of the injection of the electrochemically generated H_2_O_2_ solution. This difference between the initial rate and the rest can be attributed to the fact that there is a high concentration of organic species at the beginning of the treatment, leading to a higher removal efficacy. In addition, the fluctuation of the COD/TOC ratio (i.e., deviation from a constant) during the process was likely caused by organic matter not readily biodegradable or other recalcitrant organic contaminants in the solution.^18^

Finally, the removal efficacy observed for COD and TOC was upheld with regard to BOD elimination: the BOD decreased by 95% (91% adjusted for dilution) to reach, on average, 7.83 ± 6.49 mg O_2_/L (14.32 mg O_2_/L adjusted for dilution). The effluent met the NSF350/350-1 Class R requirement for BOD (BOD < 10 mg/L).

#### Turbidity, pH, TN, and TP

The turbidity of synthetic graywater was initially 11.2 NTU. After 90 min of treatment (Figure S3, Supporting Information), the turbidity decreased by 95%. Given that the graywater was mixed with the H_2_O_2_-containing Na_2_SO_4_ solution in equal volumes during treatment, the observed turbidity reduction of graywater was partly caused by dilution. To evaluate the influence of dilution on the turbidity reduction, synthetic graywater was diluted with Milli-Q water at a 1:1 ratio with and without continuous oxygen flow through the peroxone reactor. In the 1:1 mixture of graywater and Milli-Q water, the turbidity decreased only by 42% with constant oxygen flow and by 47% without it. The small difference in turbidity reduction with and without oxygen can be attributed to the oxygen flow favoring the suspension of particles that were prone to adhering to the reactor wall. Conversely, the vast improvement in turbidity removal during the E-peroxone process correlated with the high TOC and COD removal (*vide supra*) of this process. Small particles in suspension can be chemically oxidized and dissolve, as observed in agro-industrial wastewaters.^19^ The results indicate that the E-peroxone process reduced the turbidity of graywater effectively enough to meet the NSF 350/350–1 standard for both Class R (<5 NTU) and Class C (<2 NTU).

The pH remained stable (between 6.5 and 8) and met the NSF 350/350-1 standard (6 < pH < 9) range (Figure S4, Supporting Information). On average, the pH decreased slowly from 7.8 to 7.0 during the first hour of treatment, most likely due to intermediate organic acid production in the system from peroxone-driven oxidation reactions.^5^ The addition of the H_2_O_2_ solution, whose pH was 7.53, did not influence the pH in the peroxone reactor as synthetic graywater was composed of a variety of buffering agents such as bicarbonates. However, the constant O_2_/O_3_ flow resulted in the stripping of newly generated CO_2_ generated during mineralization of the organic constituents. Therefore, the pH rose to 7.3. A similar effect was observed by Cohen and co-workers.^20^ The change in pH was reflected in an increase of TIC (i.e., carbonate alkalinity) during the last 30 min of treatment, as shown in Figure S5 of the Supporting Information.

The electrochemical peroxone process did not readily remove TN and TP in synthetic graywater at initial concentrations of 5.10 mg/L and 3.93 mg/L, respectively. Considering the dilution of the graywater with H_2_O_2_-containing Na_2_SO_4_ solution, TN nearly remained the same, whereas TP decreased by 33% after 90 min of treatment. Moghadam et al. observed that the efficacy of phosphorus removal can be improved by favoring the peroxone reaction by increasing pH.^21^ As a result, synthetic graywater can be adjusted with higher pH before treatment, as the kinetics of ^•^OH formation are enhanced under basic conditions,^12^ for more effective phosphorus removal. TP removal could also be attributed to the removal of some suspended solids floating on top of the peroxone chamber (Kasak et al. obtained up to 44% phosphorus removal by filtering graywater).^22^ On the other hand, AOPs such as the E-peroxone process that is based on ^•^OH as the primary oxidant are known to be ineffective for nitrogen removal. For example, ammonium ion/ammonia reacts with ^•^OH at much slower rates than oxidizable contaminants.^23^ Therefore, integrating the E-peroxone process with an additional treatment step (e.g., ion exchange, activated carbon^24^ or chlorination^25^) may lead to improvement of the nitrogen removal efficiency in the case of graywater. This integration would expand the scope of application of the E-peroxone process for treating human wastewater that has higher nitrogen loads.

### 4.2 Consecutive Testing

**Figure 3:**
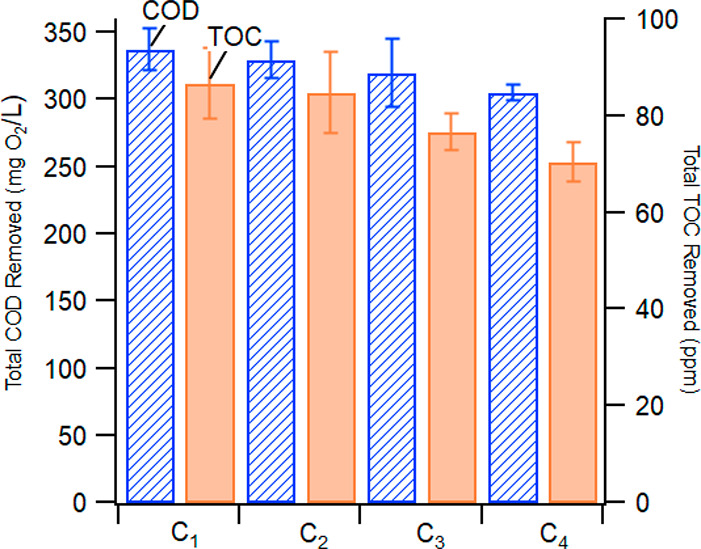
Average COD removal (COD_rem_) and TOC removal (TOC_rem_) from graywater during four consecutive treatment cycles (C_1_ through C_4_). Error bars represent ± standard deviation from the mean.

The COD removal (COD_rem_) extent remained above 300 mg O_2_/L for each 90 min treatment run (Figure 3) with a small decline from 337.25 ± 15.17 mg O_2_/L (C_1_) to 305 ± 5.7 mg O_2_/L (C_4_). Similarly, TOC removal (TOC_rem_) remained above 70 mg/L for each 90 min treatment run (Figure 3) but with a slightly more significant decline: from 86.63 ± 7.35 mg/L (C_1_) to 70.35 ± 4.2 mg/L (C_4_). Despite the decrease in TOC removal efficiency, the graywater quality standards were maintained for COD and TOC, even when the effluent was recycled into the H_2_O_2_ generator four times. The treatment performance declined faster after a fifth treatment cycle, potentially due to the decrease in electrolytic H_2_O_2_ production due to a stepwise reduction in conductivity (*vide infra*). The decline in COD_rem_ and TOC_rem_ over consecutive cycles can be explained by two main factors: (1) the impact of the recycled effluent and (2) the decrease in H_2_O_2_ production (Table S4, Supporting Information).

The COD_rem_ and TOC_rem_ decreases may also be due to the accumulation of organics in the system as the effluent from C_1_ (Ef_1_) was recycled for H_2_O_2_ electro-generation in C_2_, the value of COD Ef_1_ = 21.25 ± 15.71 mg O_2_/L and TOC Ef_1_ = 7.29 ± 7.86 mg/L (the Na_2_SO_4_ electrolyte solution used in C_0_ had no detectable amounts of COD or TOC). COD and TOC in Ef_1_ did not degrade during the electro-generation of H_2_O_2_ (data not shown). As a result, COD and TOC values increased in C_2_ compared to C_1_ when the electro-generated H_2_O_2_ solution from Ef_1_ was injected into the peroxone reactor. The same phenomenon was accentuated in the subsequent cycles (C_3_ and C_4_).

The second factor impacting COD_rem_ and TOC_rem_ decrease is the lower [H_2_O_2_] in the electro-generated solution that was injected into the peroxone reactor. This decrease is attributed to a loss of conductivity (*vide infra*). Because the peroxone reactor is saturated with O_3_, H_2_O_2_ is the limiting reagent in the peroxone process. Consequently, the decrease of [H_2_O_2_] in the solution injected into the peroxone reactor leads to a decrease of the steady state [^•^OH], which in turn impacts the degradation of organic components.

### 4.3 Effects of Recycling Treated Graywater on Electrochemical H_2_O_2_ Generation

The amount of time required to obtain an adequate [H_2_O_2_] (i.e., 4.8 mM) increased for each consecutive treatment test as treated graywater was recycled from the previous cycle for use in the electrochemical H_2_O_2_ production reactor. The increase of H_2_O_2_ generation time over multiple cycles is attributed to the change in the chemical composition of the electrolyte solution (i.e., as treated graywater is recycled). First, the solution conductivity is drastically reduced as the original 50 mM Na_2_SO_4_ electrolyte solution at a conductivity of 8 mS/cm is mixed and diluted with graywater with a much lower conductivity of ∼0.7 mS/cm to produce H_2_O_2_ in subsequent cycles. In addition, a fraction of H_2_O_2_ may be simultaneously consumed by the organic compounds remaining in treated graywater due to incomplete organic mineralization. However, if treated graywater contains a high concentrations of chloride, reactive chlorine species (RCS) including hypochlorous acid (HOCl) and hypochlorite ions will be produced at the anode^26^ during the reductive electrochemical generation of H_2_O_2_ generation, resulting in a counterproductive scavenging of the generated H_2_O_2_.^27^

**Figure 4:**
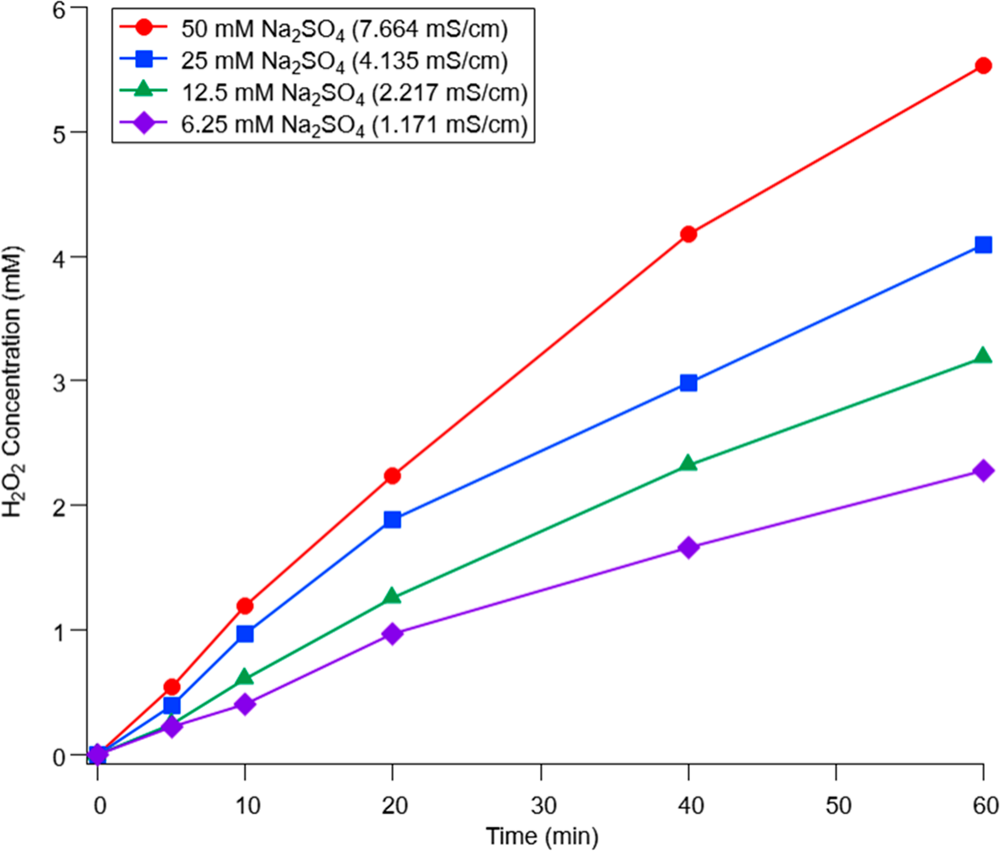
H_2_O_2_ generation in Na_2_SO_4_ electrolyte solutions at varying concentrations. H_2_O_2_ was generated for 60 min in the same setups as described in section 2.2, except that a potentiostat was used to apply 3.00 V of DC and measure the total electric charge for each electrolyte solution tested.

To reproduce the decreasing conductivity observed over the course of consecutive tests, the original 50 mM Na_2_SO_4_ electrolyte solution was diluted with Milli-Q water at varying percentages: 50%, 25%, and 12.5%. Therefore, the electrolyte conductivity decreased proportionally by the dilution factors as shown in Table 1. Isolating the conductivity as a primary variable eliminates the potential impacts of residual organics in recycled treated graywater and of RCS formation on the net production of H_2_O_2_. Figure 4 shows a decrease in H_2_O_2_ production due to decreasing conductivity in proportion to the actual Na_2_SO_4_ concentration. Lower electrolyte solution conductivity also increases the potential drop between the anode and the cathode in solution, thereby decreasing the total charge passed in the system as each test was run at a constant voltage. The Faradaic efficiency of each run was calculated based on the total moles of H_2_O_2_ generated and the total charge passed during the 60 min electrolyses. Table 1 shows that the Faradaic efficiency, which is proportional to the total moles of H_2_O_2_ produced and the inverse of total charge passed, remained unchanged regardless of the electrolyte conductivity. The constant Faradaic efficiency indicates that other factors such as electrocatalytic activity loss and parasitic side reactions^28,29^ were not present to suppress the H_2_O_2_ generation efficiency. Therefore, the electrolyte conductivity, which was reduced during the reuse of treated graywater as an influent to the first-stage reactor, was the primary factor increasing the extended time required to produce the same concentration of H_2_O_2_.

**Table 1:** Na_2_SO_4_ Electrolyte Concentration [Na_2_SO_4_]_0_, Conductivity, Final [H_2_O_2_] Produced, Total Charge Passed in Each Solution, and Faradaic Efficiency for H_2_O_2_ Generation, the Latter Calculated Based on the Final [H_2_O_2_] and the Total Charge Passed

[Na_2_SO_4_]_0_ (mM)	50.0	25.0	12.5	6.25
conductivity (mS/cm)	7.664	4.135	2.217	1.171
final [H_2_O_2_] (mM)	5.529	4.091	3.187	2.278
total charge passed (C)	812.14	612.36	475.34	314.51
Faradaic efficiency (%)	65.69	64.46	64.69	64.89

H_2_O_2_ has a high reduction potential of *E*° = 1.76 V, although less than for O_3_ (*E*° = 2.05 V) and or hydroxyl radical (*E*° = 2.85 V), which makes it a viable oxidizing agent for organic carbon in wastewater, especially when activated (e.g., UV radiation, the Fenton reaction, or by peroxidases). If the concentrations of oxidizable organic compounds in the treated graywater are high enough, then H_2_O_2_ produced in the first reactor may be consumed by the remaining organics, thereby increasing the time to achieve the target concentration of H_2_O_2_. As expected, when untreated graywater was mixed at a 1:1 volume ratio with a clean electrolyte solution, the concentration of H_2_O_2_, which was 4.8 mM at time = 0 min, decreased by 40% after 60 min of contact time (Table S5, Supporting Information). On the other hand, the loss of H_2_O_2_ was negligible in a mixture of treated graywater containing very low levels of oxidizable species after treatment with ^•^OH and O_3_ in the peroxone reactor.

If chloride is present in the influent to the first reactor, then HOCl/^–^OCl (i.e., RCS) would be produced at the anode, while H_2_O_2_ is generated at the carbon cathode. The formation of HOCl leads to direct scavenging of H_2_O_2_ as follows:^27^
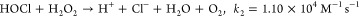
For example, when H_2_O_2_ and HOCl were combined in an equimolar molar ratio with initial concentrations of 4.8 mM, the concentration of H_2_O_2_ decreased by 30% after 60 min (Table S6, Supporting Information). However, analysis by ion chromatography (data not shown) confirmed that the concentration of chloride (<1 mM) in graywater was too low to produce a significant amount of HOCl during electrochemical H_2_O_2_ generation. The reaction of H_2_O_2_ and HOCl, however, may play a more significant role during electrochemical H_2_O_2_ generation for recycled waters with higher concentrations of chloride (e.g., toilet wastewater or brackish water).

### 4.4 Dosage of H_2_O_2_ into the Peroxone Reactor

**Figure 5:**
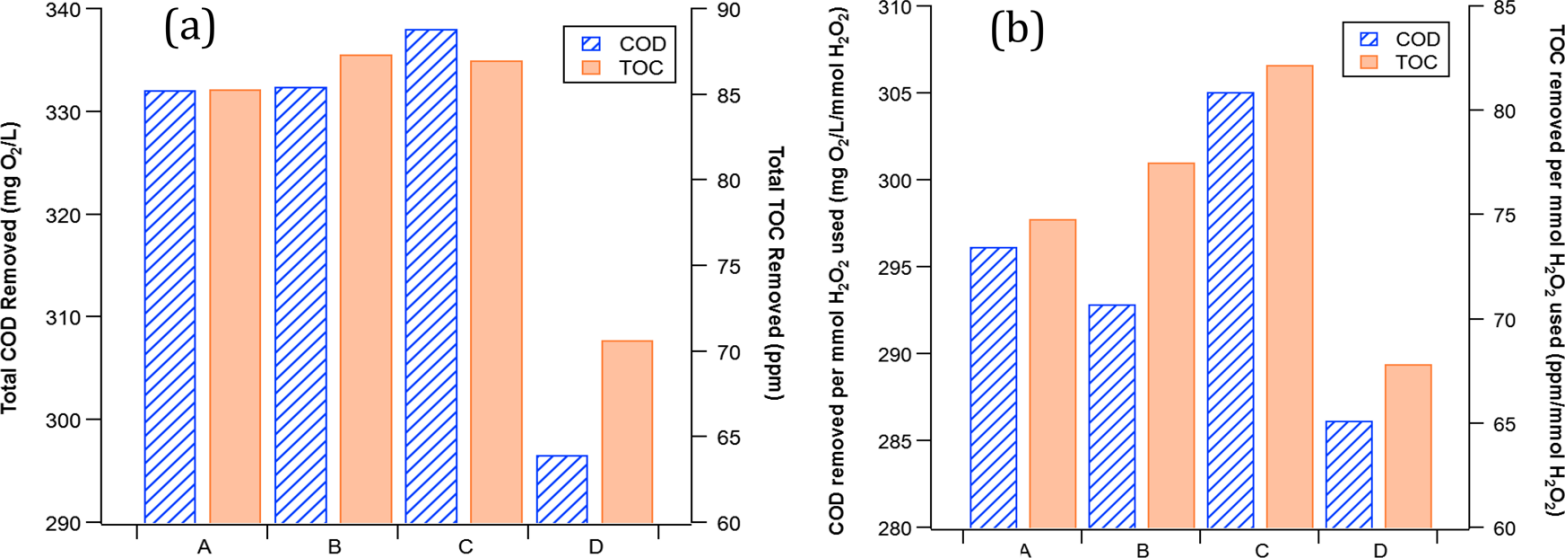
Total COD and TOC removal amounts (a) and COD and TOC removal amounts per mmol H_2_O_2_ used (b). For the 90 min treatment time frame, 250 mL of the H_2_O_2_ solution was added to the peroxone reactor completely at the beginning in experiment A, whereas three identical portions of the stage-one reactor solution were applied in the beginning and then at the 5 min and 10 min marks in experiment B, at the 10 min and 20 min marks in experiment C, and at the 20 min and 40 min marks in experiment D.

The dosage of H_2_O_2_ was determined based on the H_2_O_2_ depletion rate observed in the peroxone reactor. Excess H_2_O_2_ in the second-stage reactor will react with *in situ*^•^OH.^30,31^
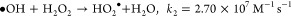
Therefore, the rate of H_2_O_2_ transfer from the first-stage reactor to the peroxone reactor is optimized such that ^•^OH reacts with the target organic molecules in graywater without being scavenged by unreacted H_2_O_2_. To maximize the use of H_2_O_2_ for the generation of ^•^OH, 250 mL of the electrochemically generated H_2_O_2_ solution was injected into the peroxone reactor in three time-separated increments during the treatment. Each 83 mL volume of the H_2_O_2_ solution was injected at the beginning of the treatment and subsequently at 5 min (experiment B), 10 min (experiment C), and 20 min (experiment D) intervals, while the control experiment (experiment A) introduced 250 mL of the electrochemically generated H_2_O_2_ solution as one addition at the beginning of the treatment experiment. The additional experimental parameters are given in section 3.1 (see above). The treatment efficacies in terms of total COD and TOC removal and treatment efficiencies in terms of COD and TOC removal per millimole of H_2_O_2_ used were examined for each experiment to optimize the H_2_O_2_ introduction intervals for more efficient use of H_2_O_2_ and to obtain a higher extent of graywater mineralization (Figure 5).

The loss of treatment efficacy, observed when the entire H_2_O_2_ solution was introduced simultaneously at the start of treatment, is reflective of unreacted H_2_O_2_ scavenging by ^•^OH, which accelerated the depletion of H_2_O_2_ in a nonproductive fashion.^30^ On the other hand, spacing the H_2_O_2_ additions during the treatment allowed for the nearly complete reaction of H_2_O_2_ with O_3_ at an optimal stoichiometric molar ratio of 1:2^32^ to produce ^•^OH before the subsequent addition. This approach led to an enhancement in treatment performance. Not only did total COD and TOC removal improve with longer intervals between H_2_O_2_ injections (Figure 5a), the COD and TOC removal efficiency per millimole of H_2_O_2_ followed the same trend (Figure 5b). However, the longer intervals between each introduction resulted in a lower H_2_O_2_ concentration than stoichiometrically needed before the following injection. As a result, the peroxone generation was impeded and therefore limited the overall treatment performance. It is important to note that optimal H_2_O_2_ addition timings may be highly dependent on the composition of wastewater to be treated. For example, wastewater with a higher COD (i.e., containing more oxidizable species) may allow for reduced intervals between each H_2_O_2_ addition and allow for ^•^OH to react with the oxidizable substrates. For the synthetic graywater used herein, 10 min intervals were found to be the optimal condition to achieve the most efficient H_2_O_2_ use and highest treatment performance.

### 4.5 Electrochemical H_2_O_2_ Generation with a PTFE-Coated Carbon Paper Cathode

Commercially available carbon-based electrodes (Table S1) were characterized and compared in terms of electrocatalytic activity for H_2_O_2_ production, chemical stability, and cost of material. Table 2 lists the 60 min averaged H_2_O_2_ production rates for each electrode of interest at varying current densities: 1 mA/cm^2^, 2 mA/cm^2^, and 5 mA/cm^2^. The applied potential for each test was located between 2 and 5 V. For each current density tested, CP75T carbon paper (AVCARB, Lowell, USA) provided the highest H_2_O_2_ production rate. Moreover, CP75T was coated with PTFE, rendering its surface invulnerable to attacks by weak acids and bases present in wastewater.^33^ The PTFE coating enhanced not only the chemical stability but also the mechanical properties of the carbon paper, including tensile strength at the break-in machine direction, which is *F*_TUCP75T_ = 20 MPa for the carbon paper electrode with PTFE coating, compared to *F*_TUCP75_ = 6.5 MPa for the carbon paper electrode without PTFE coating. As a result, CP75T was less susceptible to mechanical stress caused by the bubbling of the O_2_ flow in the H_2_O_2_ chamber than other electrodes without PTFE coating. Last, the price of the CP75T was in the middle range among all candidates ($0.6875 per cm^2^ on Fuelcellstore.com). In addition to the material of the electrodes, the optimization of the electrolyzer (e.g., hydraulic residence time of the electrolyte and interelectrode distance) can further improve H_2_O_2_ production.^34^

**Table 2:** 60 min Averaged H_2_O_2_ Production Rates for Different Cathodes As a Function of Current Density^a^

	1 mA cm^–2^ (μM/min)	2 mA cm^–2^ (μM/min)	5 mA cm^–2^ (μM/min)
CP75T	4.21	14.33	17.97
G100	0.00	0.43	15.40
C100	1.35	5.45	8.91
RVC 80 PPI	2.00	2.42	0.89
MGL 190	0.00	3.85	0.00

a More details about each material can be found in Table S1 of the Supporting Information.

**Figure 6:**
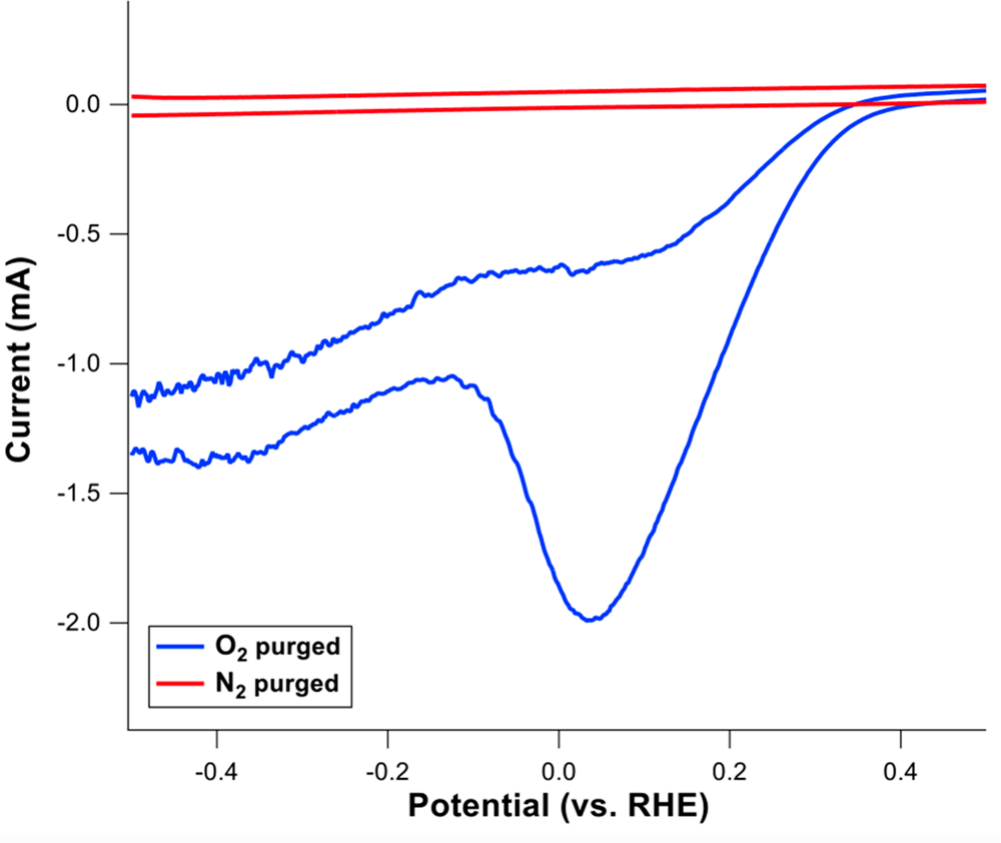
Cyclic voltammograms of PTFE-coated carbon paper electrode under oxygenated and deoxygenated conditions in 50 mM Na_2_SO_4_. Characteristic O_2_ reduction peak for H_2_O_2_ production is only visible under saturation with O_2_.

The selected PTFE-coated carbon paper was used as a cathode to confirm the H_2_O_2_ generation via O_2_ reduction by performing CV under oxygenated (O_2_ purging) and deoxygenated (N_2_ purging) conditions. Figure 6 shows cathodic currents with a reduction peak around 0.05 V vs RHE under O_2_ purging, whereas no reduction peak is visible under N_2_ purging. Therefore, the reduction of O_2_ to H_2_O_2_ took place at the cathode and appeared as the reduction peak under saturation with O_2_.^35^ The remaining cathodic currents beyond 0.05 V under the oxygenated and deoxygenated conditions are attributed to hydrogen evolution reaction.

## 5 Conclusions

The laboratory-scale treatment of synthetic graywater in an uncoupled E-peroxone process is explored in this study. By separating the *in situ* electrochemical production of H_2_O_2_ from the main reaction chamber in which ^•^OH is generated, the process not only extends the lifetime of the carbon-PTFE electrodes that are susceptible to degradation by ^•^OH but also allows for a finer control over H_2_O_2_ utilization to maximize the ^•^OH formation. Three equal volumes of the electrochemically prepared H_2_O_2_ solution were added to the main chamber in 10 min intervals to achieve the highest COD and TOC removals and the most efficient use of H_2_O_2_. The uncoupled, sequenced E-peroxone process removed 89% of COD, 86% of TOC, and 91% of BOD and provided a 95% lowering of turbidity from synthetic graywater after 90 min of treatment. The resulting effluent meets most of the requirements (e.g., COD, TOC, BOD, pH, and turbidity) established in the NSF 350/350-1 standard for the safe discharge and reuse of nonpotable water applications such as toilet and urinal flushing. Moreover, the process recycles a portion of the effluent for the subsequent electrochemical production of H_2_O_2_. The time necessary to reach [H_2_O_2_] ≈ 4.8 mM slightly increased from cycle to cycle due to decreasing solution conductivity. Nevertheless, the overall reaction process maintained removal levels above 85% (COD) and 73% (TOC) over four consecutive cycles without the addition of clean water or additional electrolyte solution into the system. The two-stage E-peroxone system can be integrated with other wastewater treatment technologies (e.g., activated carbon or chlorination) to overcome limitations of the E-peroxone process (e.g., nitrogen removal) and to allow for effective onsite treatment of human wastewater in remote areas.
