# Supplementary material for: Onsite Graywater Treatment in a Two-Stage Electro-Peroxone Reactor with a Partial Recycle of Treated Effluent
Source: ACS ES T Eng. 2021 Oct 11;1(12):1659–67. doi: 10.1021/acsestengg.1c00240 (PMC8669644; doi:10.1021/acsestengg.1c00240)
Supplement: Supplementary file 1 — ee1c00240_si_001.pdf [file ee1c00240_si_001.pdf]

Supplementary Information

Onsite Graywater Treatment in a Two-Stage Electro-Peroxone Reactor with a Partial Recycle of Treated Effluent

Léopold Dobelle[1,†], Seungkyeum Kim[2,†], Axl X Levan[3], Hugo Léandri[1], Michael R Hoffmann[1], Clément A Cid[1,*]

[1]Dept. of Environmental Science and Engineering, California Institute of Technology, 1200 E California Blvd, Pasadena, CA 91125 USA

[2]Dept. of Chemical Engineering, California Institute of Technology, 1200 E California Blvd, Pasadena, CA 91125 USA

[3]Dept. of Chemistry, California Institute of Technology, 1200 E California Blvd, Pasadena, CA 91125 USA

[*]ccid@caltech.edu

[†] the authors contributed equally to the work.

Number of pages: 9 (including this cover page)

Number of tables: 5

Number of figures: 5

Tables:

Table S1. Details about carbon-based electrode materials.

Name	Description	Supplier	Thickness (mm)	Bulk density (g/cm^3)
CP75T	Carbon paper with PTFE coating (13%)	AvCarb Material Solutions	0.255	0.33
G100	Soft Graphite Battery Felt	AvCarb Material Solutions	3.2	0.08
C100	Soft Carbon Battery Felt	AvCarb Material Solutions	3.2	0.09
RVC 80 PPI	Duocel Reticulated Vitreous Carbon (RVC) Foam	Duocel Foam	3.175	not provided
MGL 190	Molded Graphite Laminate	AvCarb Material Solutions	0.19	0.44

Table S2. Synthetic graywater recipe adapted from NSF/ANSI 350

Components	Description	Quantity in 10 L of tap water
Body Wash	Johnson's Gentle Baby Body Moisture Wash	1.59 g
Toothpaste	Colgate® Cavity Protection Toothpaste	0.159 g
Deodorant	Gillette® Clinical Clear Gel Cool Wave	0.106 g
Shampoo	Suave Essentials® Daily Clarifying Shampoo	1.007 g
Conditioner	Suave Essentials® Waterfall Mist Conditioner	1.113 g
Bath Cleaner	Lysol® Disinfectant Bathroom Cleaner	0.53 g
Hand Soap	Dial® Gold Antibacterial Hand Soap	1.219 g
Laundry Detergent	Ultra Tide® Stain Release Laundry Detergent	1.88 mL
Fabric Softener	Ultra Downy® April Fresh Liquid Fabric Softener	0.987 mL
Secondary Effluent	Obtained from the San Jose Creek Water Reclamation Plant	200 mL
L-Lactic Acid	Alfa Aesar, anhydrous, 98%	0.159 g
Na_2SO_4	Macron Fine Chemicals, anhydrous, granular, ACS grade	0.188 g
$NaHCO_3$	Macron Fine Chemicals, ACS grade	0.094 g
Na_3PO_4	Alfa Aesar, tribasic, anhydrous, technical grade	0.188 g

Table S3. Synthetic graywater characteristics and graywater test water parameters defined by the NSF 350/350-1 standard.

Parameter	Synthetic Graywater	NSF 350/350-1 Required Range
pH	7-8	6.5-8
Temperature (^{o}C)	20-22	25-35
Chemical oxygen demand (mg O_2/L)	355-362	250-400
Five-day biochemical oxygen demand (mg O_2/L)	155-162	130-180
Total organic carbon (ppm)	83-102	50-100
Total nitrogen (mg N/L)	5.10	3-5*

Total phosphorus (mg P/L)	3.93	1-3
Turbidity (NTU)	11.2[$]	50-100
E. coli (CFU/100 mL)	10^{6}[#]	10^{2}-10^{3}
Electrical conductivity (µS/cm)	700	-
Alkalinity (mg $CaCO_3$/L)	142	-

* In total Kjeldahl nitrogen.

[$] Test dust was not used in synthetic graywater recipe as the system did not have pretreatment steps such as filtration to remove mineral particles in suspension.

[#] The effluent used in synthetic graywater was obtained from the secondary clarifier of the San Jose Creek Water Reclamation Plant instead of the final clarifier as suggested by NSF/ANSI 350.

Table S4. Concentration of each H_2O_2 solution injected during the peroxone treatment from cycle 1 to cycle 4 and the conductivity of their related electrolyte before electro-generation.

	**$[H_2O_2]$ (mM)**	**Conductivity (mS/cm^2)**
Cycle 1	4.81	12.09
Cycle 2	4.80	6.52
Cycle 3	4.21	3.97
Cycle 4	3.88	2.67

Table S5. Decrease of H_2O_2 in presence of untreated graywater over an hour.

	**T (min)**				
	**0**	**15**	**30**	**45**	**60**
**Measured [H_2O_2] (mM)**	4.8	2.073	2.051	1.931	1.591
**Adjusted (mM)***	4.8	4.146	4.101	3.862	3.181
**Decrease (%)**	0	13.79%	14.74%	19.70%	33.86%

* H_2O_2 concentration in solution was adjusted for dilution to project the decrease on the initial

Table S5. Decrease of H_2O_2 in presence of HOCl over an hour

	T (min)				
	**0**	**15**	**30**	**45**	**60**
**Measured [H_2O_2] (mM)**	4.81	1.797	1.861	1.733	1.690
**Adjusted (mM)***	4.81	3.593	3.722	3.465	3.379
**Decrease (%)**	0	25.33%	22.67%	28.00%	29.78%

* H_2O_2 concentration in solution was adjusted for dilution to project the decrease on the initial

Figures:

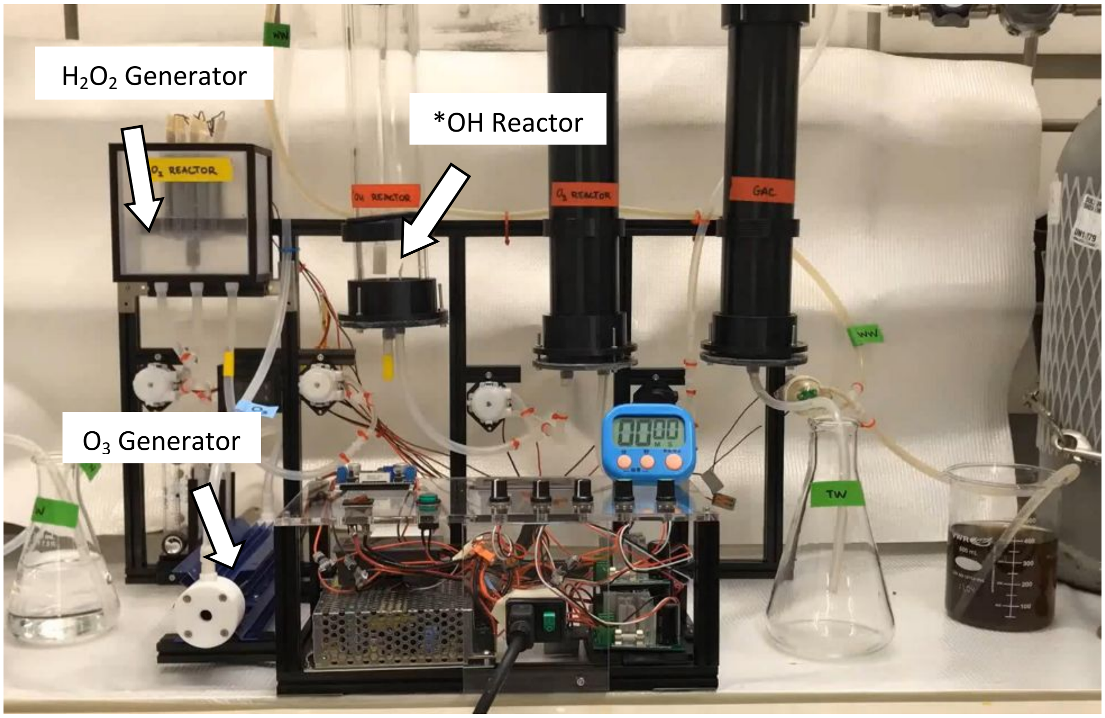

Figure S1. Laboratory-scale prototype.

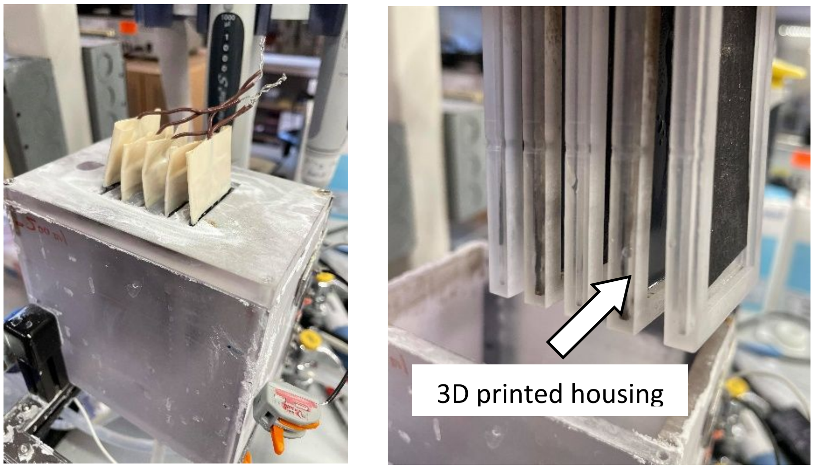

Figure S2. 3D-printed electrodes housing.

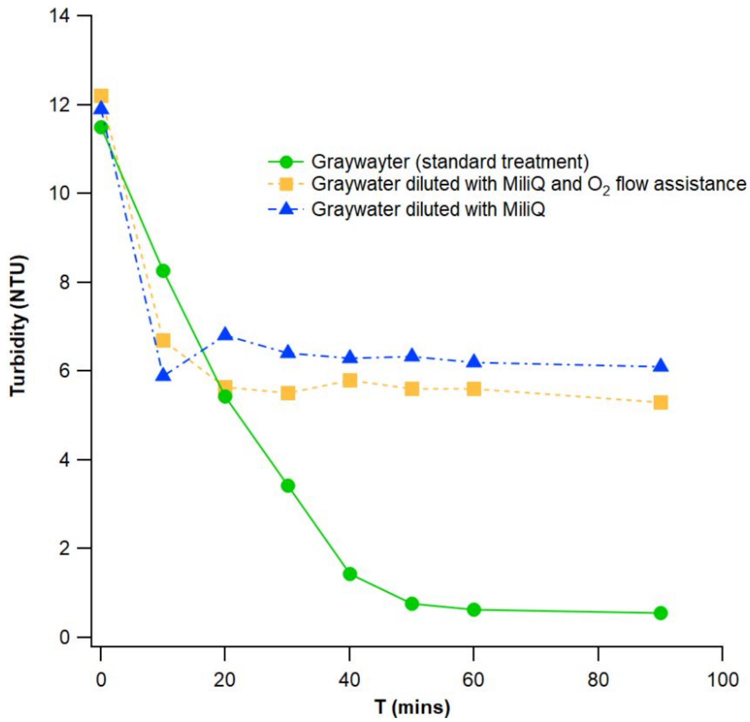

Figure S3. Turbidity trend during single-pass E-peroxone treatment of synthetic graywater. As comparison, synthetic graywater was mixed and diluted with Milli-Q water with and without O_2 sparging. Milli-Q water was added to the graywater at the same dosage as the H_2O_2-containing Na_2SO_4 solution was introduced to the *OH reactor.

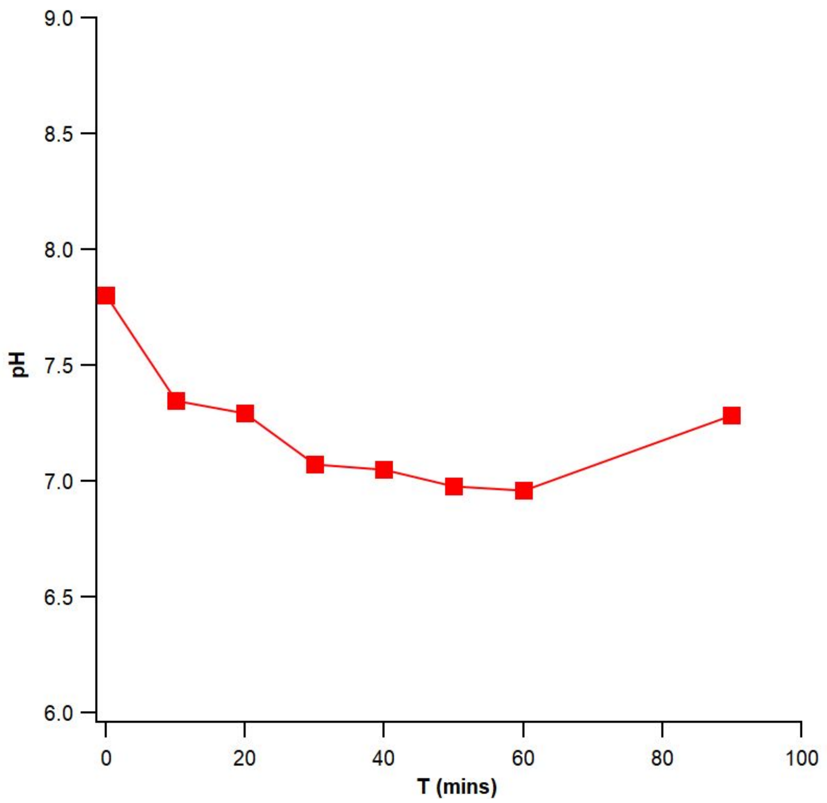

Figure S4. Evolution of pH with time during single-pass E-peroxone treatment of synthetic graywater.

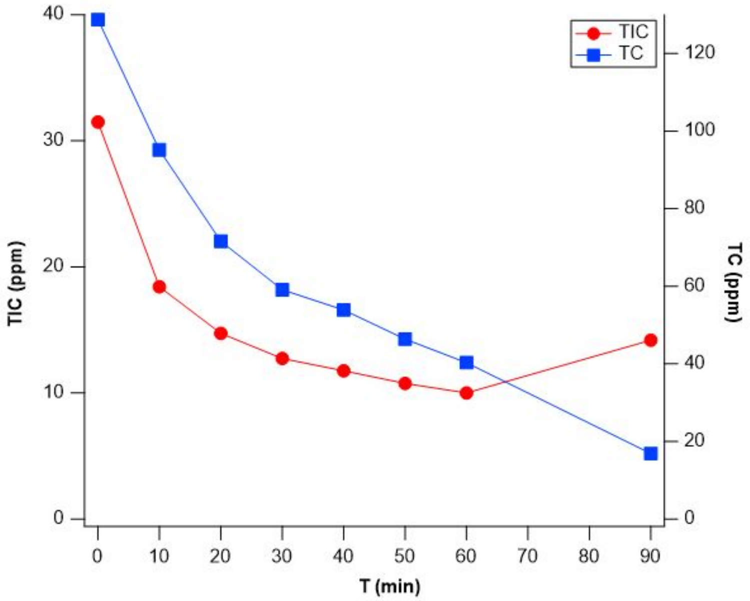

Figure S5. Total inorganic carbon (TIC) and total carbon (TC) trends during single-pass E-peroxone treatment of synthetic graywater. The TIC increased along with the pH from 60-minute to the end of the treatment while TC kept decreasing.
